# Bupropion Administration Increases Resting-State Functional Connectivity in Dorso-Medial Prefrontal Cortex

**DOI:** 10.1093/ijnp/pyx016

**Published:** 2017-03-11

**Authors:** Ewelina Rzepa, Zola Dean, Ciara McCabe

**Affiliations:** 1 School of Psychology and Clinical Language Sciences, University of Reading, UK.

**Keywords:** bupropion, antidepressant, resting-state, functional connectivity, depression

## Abstract

**Background:**

Patients on the selective serotonergic reuptake inhibitors like citalopram report emotional blunting. We showed previously that citalopram reduces resting-state functional connectivity in healthy volunteers in a number of brain regions, including the dorso-medial prefrontal cortex, which may be related to its clinical effects. Bupropion is a dopaminergic and noradrenergic reuptake inhibitor and is not reported to cause emotional blunting. However, how bupropion affects resting-state functional connectivity in healthy controls remains unknown.

**Methods:**

Using a within-subjects, repeated-measures, double-blind, crossover design, we examined 17 healthy volunteers (9 female, 8 male). Volunteers received 7 days of bupropion (150 mg/d) and 7 days of placebo treatment and underwent resting-state functional Magnetic Resonance Imaging. We selected seed regions in the salience network (amygdala and pregenual anterior cingulate cortex) and the central executive network (dorsal medial prefrontal cortex). Mood and anhedonia measures were also recorded and examined in relation to resting-state functional connectivity.

**Results:**

Relative to placebo, bupropion *increased* resting-state functional connectivity in healthy volunteers between the dorsal medial prefrontal cortex seed region and the posterior cingulate cortex and the precuneus cortex, key parts of the default mode network.

**Conclusions:**

These results are opposite to that which we found with 7 days treatment of citalopram in healthy volunteers. These results reflect a different mechanism of action of bupropion compared with selective serotonergic reuptake inhibitors. These results help explain the apparent lack of emotional blunting caused by bupropion in depressed patients.

**Significance Statement**

These results are opposite to that which we found with 7 days treatment of citalopram in healthy volunteers. These results reflect a different mechanism of action of bupropion compared with SSRIs. These results help explain the apparent lack of emotional blunting caused by bupropion in depressed patients.

## Introduction

Resting state functional connectivity (RSFC) has been found dysfunctional in patients with major depressive disorder (MDD) in networks such as the Salience Network (SN), the Central Executive Network (CEN), and the Default Mode Network (DMN) (Sheline et al., 2010).

The SN and the CEN are described as task-positive networks, activated during tasks and less active at rest in healthy controls. The SN involving the anterior insula, pregenual anterior cingulate (pgACC) and amygdala is implicated in the processing of various aspects of salient stimuli, whereas the CEN, which consists of regions such as the dorsolateral, dorsal medial prefrontal cortex, and the posterior parietal cortex, is involved in cognitive functioning including attention and working memory (Bressler and Menon, 2010a). The DMN, more active at rest, involves brain regions such as the posterior cingulate cortex, precuneus cortex, and medial prefrontal cortex and is implicated in self-referential activity and emotion regulation (Bressler and Menon, 2010b).

Abnormalities in these networks have been found in depressed patients and are thought to reflect difficulties with the suppression of unwanted thoughts, deficits in attentional control over emotional stimuli, and difficulties with emotion recognition. However there have been inconsistencies in literature regarding the direction of effects with some studies finding increased SN and CEN (Horn et al., 2010; Sheline et al., 2010; Whitfield-Gabrieli and Ford, 2012; Manoliu et al., 2014; Ramasubbu et al., 2014) whilst others find reduced connectivity (Ye et al., 2012; Tahmasian et al., 2013; Liston et al., 2014) in these regions in MDD. These inconsistencies might be related to heterogeneity in the MDD population studied and also differences in medication history. To combat problems with medication history, we recently examined young people with high depression symptomatology but no medication history. We examined seed regions in the SN and the CEN and found decreased RSFC in the symptomatic group between the amygdala and the pgACC and hippocampus and precuneus. We also found decreased RSFC in the symptomatic group between the pgACC and the putamen and between the dorso-medial prefrontal cortex (dmPFC) and the precuneus (Rzepa and McCabe, 2016). We thus suggest that networks such as the SN and CEN may be targets for normalization with treatment.

Examining the effects of pharmacological treatments on neural processes, Fu et al. report that MDD patients showed increased connectivity in the anterior DMN with 12 weeks duloxetine (selective noradrenergic reuptake inhibitor) treatment (Fu et al., 2015). However, it has recently been shown that 8 weeks treatment of escitalopram (selective serotonergic reuptake inhibitors [SSRI]) significantly reduced dmPFC activity in MDD patients after treatment, which additionally correlated with depression symptom improvement (Wang et al., 2015). The inconsistencies may be related to the change in symptoms following treatment. Thus, investigating the effects of antidepressants outside of mood changes is a useful first step in understanding their mechanism of action. We and others have examined the effects of 7 days treatment with SSRIs in healthy volunteers and found decreased functional connectivity between the prefrontal cortex and the DMN (McCabe and Mishor, 2011; McCabe et al., 2011; van de Ven et al., 2013) and decreased functional connectivity over many networks with just a single dose of an SSRI (Klaassens et al., 2015). Furthermore, 2 weeks of duloxetine (SNRI) was also found to reduce DMN and task positive network connectivity in healthy volunteers (van Wingen et al., 2014). Taken together, this suggests the direction of effects on the resting state may be different based on the mechanism of action of the antidepressants examined. Moreover, reports suggest that SSRIs can contribute to emotional blunting in patients, where experiences both positive and negative are flattened (Price et al., 2009), whereas catecholamine antidepressants like bupropion (dopamine and noradrenaline reuptake inhibitor) (Stahl et al., 2004; Dwoskin et al., 2006) might be more efficacious at improving anhedonia in depression (Dunlop and Nemeroff, 2007; Nutt et al., 2007; McCabe et al., 2009). In light of this, we recently examined the effects of 7 days treatment with bupropion and found, unlike with citalopram (SSRI), *enhanced* neural activity in healthy controls during the anticipation, effort, and consummation of rewarding and aversive stimuli (Dean et al., 2016). However, how bupropion affects RSFC remains as-yet unknown.

Thus, our current study is the first to investigate the effects of 7 days bupropion administration on the RSFC in healthy volunteers using a double-blind, placebo-controlled, crossover design. Based on previous literature, we selected regions of interest (ROIs) that have been shown to be dysfunctional in depression (amygdala, pgACC, and the dmPFC). We hypothesized that bupropion, with its opposite effects to citalopram during our fMRI tasks, might also have opposite effects on RSFC compared with citalopram. That is, we expected that bupropion might increase RSFC, compared with the placebo control condition, in line with our recent study showing increased neural activity under bupropion (Dean et al., 2016).

## Methods and Materials

### Participants

Seventeen healthy, right-handed, Caucasian volunteers (mean 24 years, 9 female) were randomized to receive 7 days oral treatment with bupropion (150 mg/d) and 7 days oral treatment with placebo separated by a 2-week washout phase in a double-blind, within-subjects design. The study was located at the Centre for Neuroscience and Neurodynamics in the Department of Psychology at the University of Reading. Volunteers were recruited via advertisement and, after reading study information, provided written consent prior to screening. Ethical approval was obtained from the University of Reading.

The exclusion criteria included current/previous psychiatric disorder (including alcohol or drug dependency) using the DSM-IV Structured Clinical Interview (First et al., 2002), pregnancy, and any contraindications to MRI and bupropion (including family history of bipolar disorder and seizures/epilepsy). Volunteers were medication free for the past 3 months (excluding the contraceptive pill) before starting the study and underwent a physical examination. Volunteers had a healthy BMI, and their liking and craving for chocolate was measured using a questionnaire (Rolls and McCabe, 2007). Eleven volunteers were nonsmokers, 4 smoked <1 cigarette/wk, 1 smoked 5 cigarettes/wk, and 1 smoked 1 to 2 cigarettes/d on average. Baseline measures of mood and anhedonia were taken using the Beck Depression Inventory (Beck et al., 1961), Snaith-Hamilton Pleasure Scale (Snaith et al., 1995), Fawcett-Clarke Pleasure Capacity Scale (FCPS) (Fawcett et al., 1983), Temporal Experience of Positive Mood (TEP) (Gard et al., 2007), and Behavioral Inhibition/Activation Scales (BIS/BAS) (Carver and White, 1994). The BIS is hypothesized to be sensitive to cues of threat or punishment and is associated with avoidant or withdrawal behaviors (Zinbarg and Lira Yoon, 2008; Bijttebier et al., 2009). The BAS is hypothesized to be sensitive to cues of reward or appetite and is associated with approach behaviors (Fowles, 1988). Given that we used taste stimuli, including chocolate, volunteers also completed the Eating Attitudes Questionnaire (Garner et al., 1982) to assess eating attitudes.

### Experimental Design

The study used a randomized, double blind, within-subjects, counterbalanced, crossover design. Volunteers received 7 days (1 tablet each morning) of bupropion treatment (150 mg/d) and 7 days of placebo treatment, separated by a 2-week washout phase. Treatment order was randomized, with 9 volunteers receiving bupropion first and 8 receiving placebo first. Volunteers underwent an fMRI scan on the 7th day of each treatment at approximately 3 hours after last dose. One volunteer had a scan after 6 days treatment (drug) due to experiencing adverse side-effects. Medication was provided by the Oxford Health NHS Foundation Trust and the Royal Free London NHS Foundation Trust. Participants were allowed only one caffeinated drink on the scan morning. Before scans, volunteers completed the Patient Rated Inventory of Side Effects (Sequenced Treatment Alternatives to Relieve Depression) to record any adverse side-effects. Mood was measured before and after scans using the befindlichkeit scale of mood and energy (von Zerssen et al., 1974) and a mood visual analogue scale (VAS).

MRI-derived measures of brain function, based on blood-oxygenation-level-dependent (BOLD) contrast, were used to compare brain responses at rest in the treatment and the placebo groups. The resting-state data were acquired before any other scans, including the structural scan. Subjects were instructed to lie in dimmed light with their eyes open, think of nothing in particular, and not to fall asleep, similar to our previous studies (McCabe and Mishor, 2011; McCabe et al., 2011; Cowdrey et al., 2012; Rzepa et al., 2015; Rzepa and McCabe, 2016) and a method found to have higher reliability than eyes closed (Patriat R et al., 2013).

### Image Acquisition

A Siemens Magnetom Trio 3T whole body MRI scanner and a 32-channel head coil were used. Multi-band accelerated echo planar imaging sequencing (Center for Magnetic Resonance Research) was used with an acceleration factor of 6 and iPAT acceleration factor of 2. T2*-weighted EPI slices were obtained every 0.7 seconds (TR=0.7, TE=0.03), these parameters were optimized given our scanner capability and used to increase sampling rates and increase our power to detect resting state networks as has been shown previously with multiband (Xu et al., 2013) (Filippini et al., 2014). Fifty-four transverse slices with in-plane resolution of 2.4 x 2.4mm were attained and slice thickness was 2.4 mm. The matrix size was 96x96 and the field of view were 230x230 mm. Acquisition was performed during resting-state scan, yielding 400 volumes in total. Sagittal 3D MPRAGE images were also acquired with an isotropic in-plane resolution of 1x1x1 (TI=0.9 seconds, TR=2.02, flip angle 9°, FOV=250x250 mm) yielding 192 slices.

### fMRI Data Analysis

#### Preprocessing

Imaging data were preprocessed and analyzed using FSL tools (www.fmrib.ox.ac.uk/fsl) (Smith et al., 2004). fMRI data preprocessing was carried out using FEAT (FMRI Expert Analysis Tool, version 6.0, a part of FSL software) and included the following steps: non-brain removal (Smith, 2002), motion correction using MCFLIRT (Jenkinson and Smith, 2001), spatial smoothing using a Gaussian kernel of full-width at half maximum of 5 mm, grand mean intensity normalization of the entire 4D dataset by a single multiplicative factor, and high pass temporal filtering (Gaussian-weighted least-squares straight line fitting, with sigma=64.0 s). fMRI volumes were registered to the individual’s structural scan and the MNI-152 standard space image (Montreal Neurological Institute, Montreal, QC, Canada) using FMRIB’s Linear Image Registration Tool (Jenkinson et al., 2001).

#### Time Series Extraction and Higher Level Analysis

To study RSFC, a seed-based correlation approach was used. Using the Harvard-Oxford subcortical structural atlas (Kennedy et al., 1998), we created a structural bilateral amygdala. To maximize the exact coverage, the masks of these seed regions were threshold by 20% to include voxels having at least 80% of probability of being in these particular regions. We also created seeds for the dmPFC (18 34 29; -24 35 28, 6-mm sphere) coordinates from (Sheline et al., 2010) and a pgACC seed (0 38 0, 8 mm sphere) coordinates from the center of the region in the Harvard-Oxford Structural atlas. Spheres were checked that they did not cross into other brain regions. The seeds were created with Wake Forest University Pickatlas tool in SPM8 similar to our recent paper (Rzepa and McCabe, 2016) and our previous RSFC paper with citalopram (McCabe et al., 2011).

The mean time course within the left and right seeds of each ROI (except for the pgACC, only comprising one medial seed) was calculated and used as a single regressor added to the general linear model. In addition, white matter signal, cerebrospinal fluid signal, 6 motion parameters (3 translations and 3 rotations), and the global mean time-series over whole brain were used as nuisance regressors. We have obtained white matter and cerebrospinal fluid masks using FSL’s FAST segmentation program. The resulting segmented images were then thresholded to ensure 80% tissue type probability. For each individual, the general linear model was analysed by using the FMRI Expert Analysis Tool [version 5.4, part of FMRIB’s Software Library (Smith et al., 2004)]. The resulting parameter estimate maps were then analyzed using higher level 1-sample *t* tests for group averages and between-samples *t* tests for group differences. Images were corrected for multiple comparisons using clusters with Z>2.3 voxel-wise thresholding and a family-wise error-corrected cluster significance threshold of *P*<.05 (Worsley, 2001). We also included FSLs FLAME1, which also considers the variance of the subject-specific parameter estimates and has been found by Eklund et al. (2016) to be a clusterwise inference that stands out as having much lower FWE, often being valid (<5%, i.e., reduced false positives) compared with other fMRI packages. From the results, we then only reported those that met the further correction for number of ROIs examined, which gave *P* < .016 (i.e., *P<*.05 Bonferroni corrected for the 3 networks of interest: amygdala, dmPFC, and pgACC) (Davidson et al., 2003). The percent BOLD signal change in the graphs is the PE/COPE values converted to mean percent BOLD signal change via Featquery (FSL; www.fmrib.ox.ac.uk/fsl;Smith et al., 2004) for the regions that had significant correlations with the seeds (Table 2).

#### Correlational Analyses

To examine the relationship between the scores on behavioral questionnaires and RSFC, we extracted the percent BOLD signal change using FSL Featquery and correlated with scores on the following questionnaires: FCPS, BIS/BAS, and TEPS.

## Results

### Demographic Details and Mood Ratings

Demographic data (Table 1) indicated that participants had low depression and anhedonia scores, as measured on range of mood and anhedonia questionnaires. A repeated-measures ANOVA was performed to examine the effect of treatment (bupropion/placebo) and time (pre-/postscan) on mood and affect, as measured by the BFS and VAS (supplementary Table 1). Results revealed that there was no significant effect of treatment [F(1,16)=.483, *P*=.497], time [*F*(1,16)=.822, *P*=.378], treatment by time [*F*(1,16)=1.922, *P*=.185], treatment by VAS [*F*(1,16)=2.472, *P*=.084] or treatment by time by VAS interactions [*F*(1,16)=.689, *P*=.545]. There was also no significant effect of treatment [*F*(1,14)=1.61, *P*=.225] or treatment by time interaction [*F*(1,14)=2.176, *P*=.162] on total BFS scores. However, there was a significant main effect of time on overall BFS score [*F*(1,14)=5.879, *P*=.029].

**Table 1.** Group Demographic and Psychosocial Measures

**Measure**	
Age (y)	24 (4.26)
Ethnicity	100% Caucasian
BMI	23.29 (2.38)
BDI	1.71 (3.14)
FCPS	136.76 (14.48)
SHAPS	20.65 (5.67)
TEPS anticipatory	47.53 (7.75)
TEPS consummatory	37.59 (4.95)
BAS drive	11.06 (2.49)
Fun seeking	11.75 (3.11)
Reward responsiveness	17.53 (1.87)
BIS	20.41 (4.24)

Abbreviations: BMI, Body Mass Index; BDI, Beck Depression Inventory; FCPS, Fawcett Clarke Pleasure Scale; SHAPS, Snaith-Hamilton Pleasure Scale; TEPS, Temporal Experience of Pleasure Scale; BAS/BIS, Behavioral Activation/Behavioral Inhibition Scale.

Data are means (SD) except for ethnicity, which is percentage.

### Adverse Effects

Supplementary table 2 reports the number of adverse effects experienced on each treatment, as measured on the Patient Rated Inventory of Side Effects. The most commonly reported adverse effects across both treatment phases were headache (n= 5 per treatment), difficulty sleeping (n= 3 per treatment), and fatigue (n= 3 placebo, n = 5 bupropion). Dizziness (n= 4) was the most commonly reported adverse effect in the bupropion condition that was not reported in the placebo condition.

### Main Effects of Stimuli on Blood Oxygen Level-Dependent Responses

Supplementary Tables 3 and 4 provide a summary of main effect of 1-sample *t* test on seed ROI functional connectivity (baseline) for the placebo and bupropion. Overall, the patterns of connectivity associated with each of the seed regions are consistent with resting-state functional connectivity experiments investigating effects of antidepressants in healthy individuals and depressed patients (Lai and Wu, 2012; Li et al., 2013; Posner et al., 2013; Wang et al., 2014, 2015; Shen et al., 2015; Sikora et al., 2016).

### Within-Subjects Analysis Examining the Treatment Effect

#### Right dmPFC Seed

**Figure 1.**
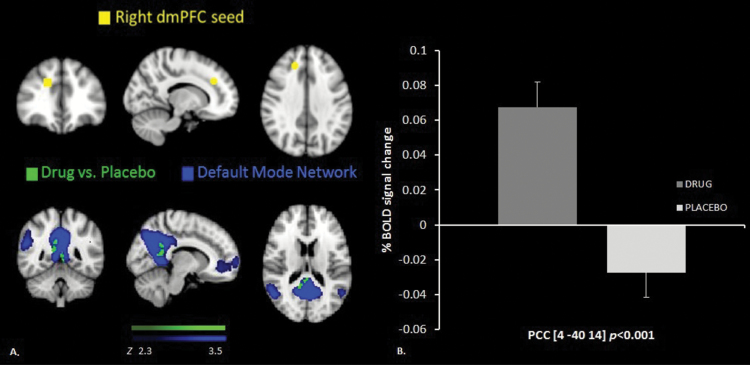
(A) Resting state functional connectivity (RSFC) between the right dorso-medial prefrontal cortex (dmPFC) seed region (yellow) and the posterior cingulate cortex, higher in the bupropion treatment than the placebo treatment (green) overlaid on the Default Mode Network (DMN). (B) Percent BOLD signal change extracted for both of the groups.

There was increased RSFC between the right dmPFC seed and the right posterior cingulate (Table 2; Figure 1) and precuneus cortex in the bupropion group compared with the placebo group. Both these results survived the correction for the number of seeds examined (*P*<.016; Bonferroni corrected for multiple comparisons). We also found increased functional connectivity between the left dmPFC seed region and the precuneus (-4 -46 48, *P*<.0394), yet this did not survive the multiple comparisons Bonferroni correction for the number of seeds examined.

**Table 2.** Resting State Functional Connectivity for the dmPFC Seed Region

	**MNI coordinates**					
**Brain Region**	**X**	**Y**	**Z**	**Cluster size**	***z*-Score**	***P* value**
dmPFC seed bupropion > placebo						
PCC Precuneus	4 8	-40 -58	14 32	449 449	3.47 3.1	^<0.001^ ^<0.001^

*P* values cluster corrected family wise error *P* < .05, further corrected for multiple comparisons across 3 seed networks resulting in *P* = .016.

PCC, posterior cingulate cortex.

#### Correlations with Behavioral Measures

We found a negative correlation between the decreased RSFC of the dmPFC seed and the precuneus and increased BIS under the drug condition (r=-.494, *P*=.044; however, this did not survive multiple correction for the number of questionnaires examined) and no correlation in the placebo condition (r=.404, *P*=.108).

**Figure 2.**
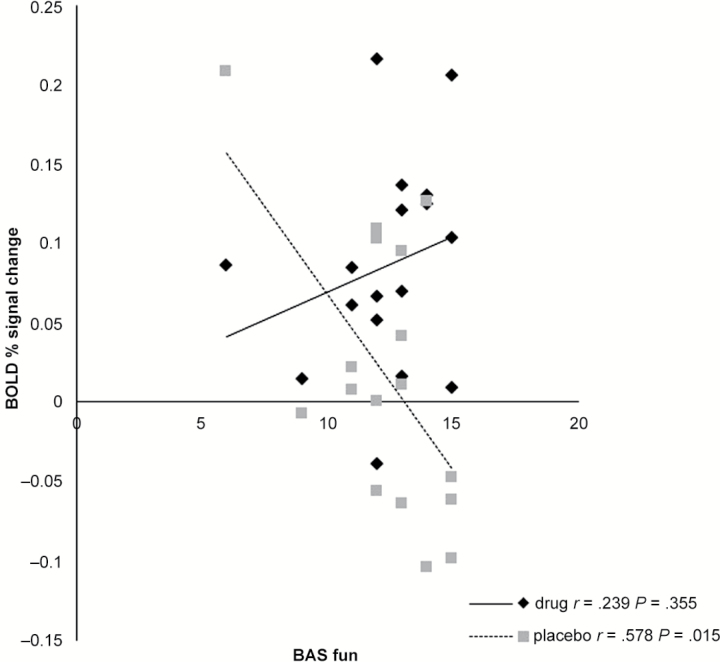
Decreased Resting state functional connectivity (RSFC) between the dorso-medial prefrontal cortex (dmPFC) region and the precuneous correlates with decreasing Behavioral Activation Scale (BAS) fun scores under placebo only.

We also found a negative correlation between decreased RSFC of the dmPFC seed and the precuneus and decreased behavioral activation subtype of fun (BAS fun) under the placebo condition (r=-.578, *P*=.015; this also survived multiple correction for the number of questionnaires) but no correlation in the drug condition (r = .239, *P*=.355) (Figure 2). We also examined the difference in these correlations with the Fishers r to z transformation and found z= -2.68, *P<*.007 (2-tailed). This means that there was decreased connectivity in the placebo group as levels of fun reported decreased.

## Discussion

The main aim of our study was to investigate the effects of 7 days bupropion treatment on RSFC in healthy volunteers. Our previous work found that the serotonin reuptake inhibitor citalopram reduced not only the brain’s activity during reward and aversive processing but also reduced RSFC (McCabe et al., 2010, 2011; McCabe and Mishor, 2011). We suggested that this profile of action might be related to citalopram’s positive clinical effects but perhaps also emotional blunting. Drugs like bupropion, which are dopaminergic noradrenergic reuptake inhibitors, are thought less likely to cause emotional blunting. In keeping with this notion, we have recently shown that bupropion (unlike citalopram) can increase the brain’s activity during the processing of positive and negative stimulation (Dean et al., 2016). Therefore, we hypothesized that perhaps bupropion would also increase the brain’s RSFC in key regions such as the dmPFC that we found modulated by citalopram (McCabe et al., 2011).

 Our main findings revealed that bupropion *increased* RSFC between the dmPFC seed region and the PCC and between the dmPFC and the precuneus cortex compared with placebo. The dmPFC is implicated in many cognitive and emotional processes, while the precuneus and the PCC are thought to be involved in self-referential and memory processes (Bressler and Menon, 2010b; Jaworska et al., 2015; Janssen et al., 2015). Studies of RSFC in depressed patients have revealed abnormalities in similar regions (Abbott et al., 2013; Manoliu et al., 2013; Zhong et al., 2016), with the authors suggesting that these abnormalities might represent patients’ difficulty in disengaging from self-referential processes, which might lead to increased negative thoughts (Manoliu et al., 2013). Furthermore, van Tol et al., 2014 report decreased cortical thickness related to decreased RSFC between the dmPFC and the PCC/precuneus cortex in depressed patients. The authors suggest this might lead to maladaptive emotional regulation. Thus, our finding of increased RSFC between the dmPFC and the precuneus, key regions found dysfunctional in depression, might help explain the mechanism of action of treatments like bupropion. Moreover, we also found that our results are consistent with our previous data in young, unmedicated participants with depression symptoms that revealed reduced functional connectivity between the dmPFC and the precuneus (Rzepa and McCabe, 2016). Having found the same functional connectivity now increased under the bupropion condition suggests that perhaps these networks are involved in the putative mechanism of action of bupropion. Further studies should clarify this in depressed patients before and after treatment.

Interestingly, we also found a relationship between the BIS and the dmPFC precuneus RSFC in the bupropion group and the BAS in the placebo group. The BIS is hypothesized to be sensitive to cues of threat or punishment and is associated with avoidant or withdrawal behaviors (Zinbarg and Lira Yoon, 2008; Bijttebier et al., 2009). The BAS is hypothesized to be sensitive to cues of reward or appetite and is associated with approach behaviors (Fowles, 1988). Studies in depressed patients have shown high levels of BIS activation and lower levels of BAS activation (Fowles, 1988; Gray, 1994; Davidson, 1998;Meyer et al., 2001; Kasch et al., 2002; Johnson et al., 2003; Campbell-Sills et al., 2004; Pinto-Meza et al., 2006).

In this study, we found increased RSFC under the drug condition (between the dmPFC and the precuneus) that correlated with reduced behavioral inhibition scores. However, this result did not survive the further Bonferroni correction for the number of questionnaires examined. Nonetheless, we believe it would be of interest to examine in future larger studies how bupropion might affect the avoidance of negative information. Interestingly though, a recent study examining internet gaming addiction found that 6 weeks of bupropion treatment had a greater improvement on BIS scores than the SSRI escitalopram (Song et al., 2016).

As mentioned above, we also found that as RSFC under the placebo condition was increased (between the dmPFC and the precuneus), behavioral activation (Fun) levels were also higher. This result suggests that increased RSFC between these regions is correlated with increased feelings of pleasure and supports the notion that increasing connectivity under bupropion would be unlikely to cause emotional blunting (the flattening of emotional experiences). However, as there was no significant effect under the bupropion condition, this is purely speculative and would need to be examined with a larger sample size in healthy volunteers and then again in a depressed patient sample both before and after treatment.

Taken together, our results find that bupropion increases RSFC between the dmPFC seed region and the posterior cingulate cortex and the precuneus. The RSFC with the precuneus also correlates with increased experience of fun in the placebo condition. The RSFC results are opposite to that which we found previously with citalopram using similar methodology and seed regions and opposite to that found in young unmedicated people with depression symptoms. Thus, our results may indicate a mechanism by which bupropion might work as an antidepressant by increasing dmPFC RSFC.

## Supplementary Material

Supplementary data are available at *International Journal of Neuropsychopharmacology* online.

## Funding

This work was supported by the University of Reading fund for Dr McCabe.

## Statement of Interest

Dr. McCabe has acted as a consultant to P1Vital, Givaudan, GWpharma, the British Broadcasting Company, and Channel 4. Zola Dean, Ewelina Rzepa, and Zola Dean report no biomedical financial interests or potential conflicts of interest.

## Supplementary Material

Supplementary Data
