# Supplementary material for: Bupropion Administration Increases Resting-State Functional Connectivity in Dorso-Medial Prefrontal Cortex
Source: Int J Neuropsychopharmacol. 2017 Mar 11;20(6):455–62. doi: 10.1093/ijnp/pyx016 (PMC5458340; doi:10.1093/ijnp/pyx016)
Supplement: Supplementary Data [file pyx016_suppl_Supplementary_Data.docx]

**Supplementary Data**

Bupropion administration increases resting state functional connectivity in dorso-medial prefrontal cortex.

**Running title**:

Category:

The effects of bupropion on resting-state functional connectivity.

Regular research article

Ewelina Rzepa MSc, Zola Dean MSc, Ciara McCabe* PhD.

School of Psychology and Clinical Language Sciences, University of Reading, UK.

* Corresponding author:

**Dr Ciara McCabe**

Associate Professor in Neuroscience,

School of Psychology and Clinical Language Sciences,

University of Reading,

Reading RG6 6AL,

Tel: +44 118 378 5450

[c.mccabe@reading.ac.uk](mailto:c.mccabe@reading.ac.uk)

**Table S1.** Subjective state ratings pre- and post-scan after 7 days of treatment with bupropion and placebo, separated by a two-week washout phase. There were no significant effects of treatment condition on any of the measures.

| **Measure** | **Bupropion**  **Mean (SD)** | | **Placebo**  **Mean (SD)** | |
| --- | --- | --- | --- | --- |
|  | Pre-scan | Post-scan | Pre-scan | Post-scan |
| **VAS** |  |  |  |  |
| Alertness | 5.35(2.05) | 3.67 (2.66) | 6.18 (2.38) | 5.85 (7.56) |
| Disgust | 1.39 (1.27) | 1.79 (1.86) | 0.92 (1.22) | 1.69 (1.71) |
| Drowsiness * | 2.96 (2.49) | 3.89 (2.83) | 1.73 (1.56) | 4.03 (2.53) |
| Anxiety * | 2.05 (2.03) | 0.90 (0.74) | 1.34 (1.38) | 0.80 (0.89) |
| Happiness ^*^ | 6.18 (1.61) | 5.00 (2.68) | 6.65 (1.76) | 5.68 (2.29) |
| Nausea | 0.61 (0.76) | 1.29 (2.20) | 0.65 (0.90) | 1.28 (2.05) |
| Sadness | 0.91 (0.78) | 0.92 (1.35) | 0.67 (0.52) | 0.98 (1.90) |
| Withdrawn | 0.93 (0.92) | 1.55 (1.79) | 1.15 (1.26) | 1.15 (0.99) |
| Faint * | 0.54 (0.66) | 1.58 (1.95) | 0.87 (1.25) | 1.61 (1.95) |
| **Total BFS score *** | 18.20 (11.03) | 21.20 (12.63) | 12.33 (9.80) | 19.33 (12.51) |

SD, standard deviation; VAS, visual analogue scale (*n*=17); BFS, the Begindlischskeit Scale (*n*=15); Repeated measure analyses of variance; *p*>0.05; *Significant main effect of time (pre/post scan) *p<*0.05.

**Table S2.** Frequencies of Adverse Effects Reported Under Bupropion (*n*=17) and Placebo (*n*=17).

| **Adverse Event** | **Placebo** | **Bupropion** |
| --- | --- | --- |
| **Gastrointestinal symptoms** |  |  |
| Diarrhoea | 2 | 1 |
| Constipation | 1 | 0 |
| Dry Mouth | 0 | 2 |
| Nausea or vomiting | 1 | 1 |
| **Heart symptoms** |  |  |
| Palpitations | 0 | 1 |
| Dizziness when standing | 1 | 1 |
| Chest pain | 0 | 1 |
| **Skin symptoms** |  |  |
| Rash | 1 | 1 |
| Increase perspiration | 0 | 0 |
| Itching | 1 | 0 |
| Dry skin | 2 | 1 |
| **Nervous system symptoms** |  |  |
| Headache | 5 | 5 |
| Tremors | 0 | 0 |
| Poor coordination | 0 | 1 |
| Dizziness | 0 | 4 |
| **Eye and ear symptoms** |  |  |
| Blurred vision | 1 | 0 |
| Ringing in the ears | 0 | 0 |
| **Genital and urinary symptoms** |  |  |
| Difficulty urinating | 1 | 0 |
| Painful urination | 0 | 1 |
| Frequent urination | 0 | 1 |
| Menstrual irregularity | 0 | 0 |
| **Sleep symptoms** |  |  |
| Difficulty sleeping | 3 | 3 |
| Sleeping too much | 3 | 0 |
| **Sexual functioning** |  |  |
| Loss of sexual desire | 0 | 1 |
| Trouble achieving orgasm | 0 | 0 |
| Trouble with erections | 0 | 0 |
| **Other symptoms** |  |  |
| Anxiety | 0 | 2 |
| Fatigue | 3 | 5 |
| Poor concentration | 1 | 1 |
| Decreased energy | 1 | 3 |
| General malaise | 0 | 1 |
| Restlessness | 0 | 1 |
| **Other**  Decreased appetite  Increase Energy  Irritable | 0  0  0 | 1  1  1 |

Data are frequencies.

Table S3: Regions showing significant effect of placebo for the selected seed regions.

| MNI coordinates | | | | | |
| --- | --- | --- | --- | --- | --- |
| Brain region | X | Y | Z | p-value | z-value |
| ***Left amygdala seed*** |  |  |  |  |  |
| Amygdala | -20 | -4 | -20 | <.001 | 8.26 |
| Temporal pole | -36 | 4 | -20 | <.001 | 4.34 |
| Parahippocampal gyrus | 22 | -20 | -18 | <.001 | 3.89 |
| Temporal pole | 40 | 6 | -22 | 0.0074 | 3.41 |
| Insula | -38 | -2 | 12 | 0.044 | 3.07 |
| Putamen | -28 | 4 | 10 | 0.044 | 2.82 |
| ***Right amygdala seed*** |  |  |  |  |  |
| Amygdala | 24 | -6 | -16 | <.001 | 8.26 |
| Temporal pole | 24 | 6 | -22 | <.001 | 5.83 |
| Parahippocampal gyrus | -18 | -26 | -14 | <.001 | 4.39 |
| Temporal fusiform gyrus | -38 | -14 | -28 | <.001 | 3.31 |
| MFG | 0 | 42 | -20 | 0.0024 | 3.58 |
| Frontal Pole | -6 | 58 | -6 | 0.0024 | 3.11 |
| Central Opecular cortex | 56 | -12 | 8 | 0.026 | 3.33 |
| Planum Temporale | 66 | -18 | 10 | 0.026 | 3.05 |
| ***Left dmPFC seed*** |  |  |  |  |  |
| dmPFC/Frontal pole | -22 | 34 | 26 | <.001 | 7.6 |
| Frontal Pole | -24 | 46 | 28 | <.001 | 5.76 |
| Paracingulate gyrus/ACC | 0 | 34 | 26 | <.001 | 4.94 |
| ACC | -2 | 16 | 24 | <.001 | 4.16 |
| Frontal pole | 24 | 48 | 20 | <.001 | 4.82 |
| Precuneus | -6 | -44 | 46 | <.001 | 4.31 |
| PCC/ACC | -4 | -24 | 36 | <.001 | 4.01 |
| SFG | -20 | 12 | 56 | 0.009 | 3.31 |
| MFG | -28 | -2 | 58 | 0.009 | 3.15 |
| Precuneus/Cuneal Cortex | -4 | -80 | 40 | 0.018 | 4.35 |
| Insula | 36 | 18 | 2 | 0.03 | 3.43 |
| ***Right dmPFC seed*** |  |  |  |  |  |
| dmPFC/Frontal pole | 18 | 34 | 32 | <.001 | 6.25 |
| MFG | 30 | 32 | 34 | <.001 | 4.36 |
| Paracingulate Gyrus | 10 | 50 | 14 | <.001 | 3.97 |
| ***pgACC*** |  |  |  |  |  |
| pgACC | 0 | 38 | 2 | <.001 | 8.41 |
| ACC  Frontal pole  PCC/ACC  PCC/Precuneus  OFC  Temporal pole  OFC/Insula  Temporal pole  MTG  MTG  Parahippocampal gyrus  Hippocampus  Lateral Occipital Cortex | 6  6  -4  2  -36  -44  34  46  62  -60  -18  -20  -50 | 30  56  -20  -42  14  16  18  20  0  -24  -22  -14  -66 | 8  4  42  38  -18  -16  -18  -18  -16  -8  -22  -20  24 | <.001  <.001  <.001  <.001  <.001  <.001  <.001  <.001  <.001  <.001  0.005  0.005  0.014 | 5.75  4.88  4.15  4.1  4.43  3.84  4.45  4.39  4.33  4.03  4  3.45  3.63 |
|  |  |  |  |  |  |

P<0.05 whole brain cluster corrected (Family wise error for multiple comparisons)

dmPFC- dorsal medial prefrontal cortex, OFC- orbitofrontal cortex; PCC- posterior cingulate cortex; ACC- anterior cingulate cortex; pgACC- pregenual anterior cingulate cortex; MFG- middle frontal Gyrus; SFG- superior frontal gyrys; STG- superior temporal gyrus

| Table S4: Regions showing significant effect of placebo for the selected seed regions. | | | | | | |
| --- | --- | --- | --- | --- | --- | --- |
| MNI coordinates | | | | | | |
| Brain region | | X | Y | Z | p-value | z-value |
| ***Left amygdala seed*** | |  |  |  |  |  |
| Amygdala | | -24 | -4 | -20 | <.001 | 8.07 |
| Temporal pole | | -32 | 4 | -26 | <.001 | 4.43 |
| Thalamus | | 6 | 0 | 0 | <.001 | 3.99 |
| Insula | | -38 | 0 | -14 | <.001 | 3.89 |
| STG | | 56 | 4 | -16 | 0.0026 | 3.42 |
| Temporal Pole | | 46 | 8 | -18 | 0.0026 | 3.41 |
| *Parahippocampus* | | 18 | 0 | -36 | 0.0026 | 2.93 |
| MTG | | -58 | -8 | -14 | 0.021 | 3.59 |
| ***Right amygdala seed*** | |  |  |  |  |  |
| Amygdala | | 20 | 0 | -20 | <.001 | 8.06 |
| Temporal Pole | | 46 | 10 | -14 | <.001 | 3.52 |
| Postcentral gyrus | | 44 | -18 | 40 | <.001 | 3.72 |
| MTG | | 64 | -40 | 8 | 0.036 | 3.53 |
| ***Left dmPFC seed*** | |  |  |  |  |  |
| Frontal Pole/dmPFC | | -22 | 36 | 26 | <.001 | 7.02 |
| MFG | | -30 | 34 | 30 | <.001 | 6.41 |
| ACC | | 0 | 22 | 34 | <.001 | 5.1 |
| Frontal Pole | | -26 | 50 | 22 | <.001 | 4.84 |
| Paracingulate Gyrus | | 0 | 14 | 42 | <.001 | 4.14 |
| Precuneus | | -2 | -50 | 46 | <.001 | 4.45 |
| Precuneus | | 6 | -48 | 48 | <.001 | 4.04 |
| SFG | | -24 | -2 | 66 | <.001 | 4.05 |
| Insula | | -34 | 16 | 4 | <.001 | 4.16 |
| ***Right dmPFC seed*** | |  |  |  |  |  |
| Frontal Pole | | 18 | 34 | 30 | <.001 | 6.33 |
| Paracingulate gyrus | | 10 | 44 | 32 | <.001 | 4.31 |
| MFG | | 30 | 20 | 32 | <.001 | 4.01 |
| Precuneus/PCC | | 4 | -54 | 12 | <.001 | 4.29 |
| Precuneus/PCC | | -2 | -48 | 12 | <.001 | 3.66 |
| SFG | | -10 | -52 | 24 | <.001 | 3.45 |
| Paracingulate Gyrus | | 0 | 54 | 4 | <.001 | 4.13 |
| Paracungulate gyrus/ACC | | 10 | 48 | 6 | <.001 | 3.71 |
| Paracungulate gyrus/ACC | | -12 | 48 | 6 | <.001 | 3.65 |
| MTG | | 60 | -12 | -14 | 0.018 | 3.72 |
| Medial frontal cortex | | -2 | 52 | -16 | 0.019 | 3.32 |
| Medial frontal cortex | | 4 | 42 | -12 | 0.019 | 3 |
|  |  |  |  |  |  |  |
|  |  |  |  |  |  |  |
| ***pgACC seed*** | |  |  |  |  |  |
| pgACC | | 0 | 38 | 2 | <.001 | 7.84 |
| sgACC | | 0 | 24 | -6 | <.001 | 5.78 |
| Paracingulate gyrus | | 8 | 54 | 4 | <.001 | 5.14 |
| ACC | | -2 | 36 | 22 | <.001 | 5.04 |
| PCC/Precuneus | | 10 | -46 | 36 | <.001 | 4.37 |
| MTG | | 60 | -6 | -18 | <.001 | 4.58 |
| OFC | | 40 | 26 | -22 | <.001 | 4.48 |
| Temporal Pole | | 56 | 8 | -26 | <.001 | 3.9 |
| Lateral Occipital cortex | | -60 | 66 | 28 | <.001 | 3.92 |
| Brain stem | | 6 | -48 | -44 | 0.002 | 4.13 |
| Parahippocampal Gyrus | | 22 | -24 | -16 | 0.01 | 4.63 |
| Hippocampus | | 30 | -16 | -24 | 0.01 | 2.92 |
|  | |  |  |  |  |  |

P<0.05 whole brain cluster corrected (Family wise error for multiple comparisons)

dmPFC- dorsal medial prefrontal cortex, OFC- orbitofrontal cortex; PCC- posterior cingulate cortex; ACC- anterior cingulate cortex; pgACC- pregenual anterior cingulate cortex; sgACC-subgenual anterior cingulate cortex; MFG- middle frontal Gyrus; MTG- middle temporal Gyrus; SFG- superior frontal gyrys; STG- superior temporal gyrus
